# Editorial Notes

**Published:** 1869-05-15

**Authors:** 


### EDITORIAL NOTES.

We published, in No. 3 (Feb. 1st.) of the JOURNAL, a translation of the splendid generalization of Dr. Rabuteau, termed, by him, the *Thermic Law*, and its application to the *biatomic metalloids*. Dr. Rabuteau has extended his investigations upon this subject to the *monoatomic metalloids*. This law, as applied by Dr. Rabuteau to the former group of bodies, and determining for them a therapeutic activity in direct ratio to their atomic weights, or, what is the same thing, in the inverse ratio to their specific heat, had been already applied to certain of the latter, by Bouchardat and Stuart Cooper, who discovered that chlorine is more active than bromine, and the latter than iodine. M. Rabuteau has demonstrated that fluorine satisfies this law, for the fluorides are the most active of all the monoatomic metalloid compounds. He has determined, moreover, that the fluorides are not so dangerous as had been imagined, for one grain of the fluoride of sodium having been injected into the veins of a dog, produced only transitory effects, consisting in a very marked febrile reaction and abundant salivation. M. Rabuteau having taken twenty-five centigrammes of fluoride of sodium, experienced only slight epigastric uneasiness. The insoluble fluorides, such as those of calcium of zinc or lead are inoffensive. The fluoride of barium, a slightly soluble salt, is poisonous, not on account of the fluorine which it contains, but of the barium, which is poisonous. The fluoride of strontium is much less active than the preceding salt. The most dangerous compounds of fluorine examined by the author are those of silver, and of hydrogen (hydro-fluoric acid). These compounds are painful caustics and the cicatrization of the part to which they are applied ensues only at the end of three weeks.

We shall take occasion, hereafter, to refer to the author's observations upon the compounds of iodine, bromine and chlorine, which, at this moment, possess peculiar interest for practical men, in view of their increasing popularity and extensive application as therapeutic agents.

The investigations of M. Hermann into the chemical composition of muscle, and the chemical modification which muscular fibre undergoes during contraction, have induced him to give to the electrical phenomena which take place in nerves and muscles, an interpretation based solely upon the chemical changes which take place in these tissues.

M. Hermann asserts that muscle in its normal state always contains a certain very complete nitrogenous substance dissolved in the muscular plasma. This subtance, called *inogene*, may be decomposed and form a new product, amongst other carbonic acid, sarcolactic [illegible] albuminoid body, which is separated first und[illegible] and which subsequently becomes solid [illegible]

* Etude expérimentatale sur les effets physiolo[illegible] composes metalliques en general. Paris, 1857.—[illegible]

This decomposition of inogene occurs spontaneously but very slowly in the state of rest; it occurs immediately under the influence of excitants of muscular contractility. The active state of muscle is due exactly to this rapid decomposition of inogene, aud this substance once exhausted, muscular activity is no longer possible.

This substance has not yet been isolated, because the least chemical manipulation decomposes it. Regarding its composition it may be placed by the side of hæmato-globuline, for during decomposition only they both afford an albuminoid substance.

In nerves there should be an analogous substance, which should undergo decomposition with equal rapidity, but without occasioning the formation of myosine. From these chemical data M. Hermann establishes the two following principles:

1st. If in a nervous or muscular fibre, there are points at which the decomposition of this fundamental substance occurs more rapidly than at others; the former possess, relatively to the latter, negative electricity, and the currents will be so much the stronger as the difference in decomposition is greater.

2d. The decomposition which occurs in nerves and muscles is as much more slow during the active state of these tissues, as it is more rapid during the state of repose.

The first proposition serves to explain why muscular currents are formed between the horizontal surface of a muscle or of a nerve and its artificial transverse surface, for the portion denuded and exposed to the air exhibits a rapidity of decomposition greater than the portion intact; so the transverse section is negative by relation to the longitudinal sect[illegible] sitive.

[illegible] rts another general fact, that, of unin-[illegible] aller extremity is negative in compar-[illegible] ut of the sartorius muscle the inferior [illegible] smaller than the superior, which is negative. The same is true of the semi-membranosus, whose least voluminous extremity is positive. These conclusions of M. Hermann are contested by M. DuBois-Reymond, and the discussion between these two prominent authorities is criticised by M. Onimus in Robin's *Jour de l'Anatomie et de la Physiol.*

M. Legros and Onimus having concluded a very extensive series of experimental investigations into the subject of the movements of the intestine, submit the following conclusions:

Inspiration and expiration induce in the intestine changes of tension which vary with the mode of respiration.

The movements proper to the intestine are of three sorts: 1st. the peristaltic movement, which is normal; 2d. the antiperistaltic movement; 3d. contraction.

Every peristaltic movement is characterized by an abrupt contraction, followed by a distension of longer duration.

Isolated peristaltic movements may occur in a restricted portion of the intestinal tract. After a series of contractions there is generally a period of prolonged repose.

Contractions are most frequent in the upper portion of the intestinal canal. We have never observed more than eighteen of them per minute.

The contractions of the large intestine differ from those of the small by their amplitude, their duration and their form.

The diagram of the act of defacation is entirely special, and suggests that obtained after poisoning with strychnia. The movements of the stomach have not the regularity of those of the intestine. They are of two sorts according as they are observed at the pyloris or at the great cul-de-sac.

The antiperistaltic movement does not combine with thə peristaltic.

The peristaltic movement is due to the action of the nervous cells of the intestinal walls upon the smooth fibres.

These cells may themselves undergo the influence of the caliac plexus and of the cerebro-spinal centres.

Arrest of the arterial circulation augments or determines peristaltic contractions. Arrest of the venous circulation induces no analogous effects.

This pathological condition cannot serve to explain normal movements.

Induction currents applied directly to the insestines cause contraction at the level of the poles; between the poles there is relaxation of the walls.

Continuous currents prevent peristaltic movements, and induce a diminution of tension if the current follows the normal direction of the movements, or an augmentation, if the currents pass in the opposite direction. Electrization of the spinal cord by continuous currents augments, perceptibly, the peristaltic contractions at the moment of their application.

Induction currents upon the splanchnic nerves augment, progressively, the tension, without determining peristaltic movements.

Continuous currents upon the splanchnic nerves occasion peristaltic contractions. Electricity directed upon the nervous plexi and upon the mesenteric nerves produces analogous effects.

Interrupted currents upon the pneumogastric cause dilatation and immobility of the intestine. This phenomenon occurs through reflex action. They iuduce, on the contrary, directly, contraction of the stomach.

Continuous currents, directed upon the pneumogastric, act but little upon the intestine. They arrest normal or pathological contractions of the stomach.

Direct excitation of the intestine, by pinching or otherwise, induces, at the first instant, distention followed by energetic contraction.

Ice-water arrests peristaltic movements, inducing contraction; hot water intensifies them.

Water holding in solution chloride of sodium, increases the energy of the movements.

Croton-oil determines contraction, in addition to those for the expulsion of matters; ipecacuanha acts in an analogous manner.

Saline purgatives do not increase the energy of the contractions.

Atropine, in weak doses, increases the peristaltic movements, but in strong doses abolishes them.

Morphine retards the contractions without abolishing them.

Strychnia, in poisonous doses, determines spasmodic contractions, when there are general convulsions.

From a review in Robin's *Journal de l'Anatomie et dex Animaux* of recent experimental investigations upon the innervation of the sphincters of the anus and bladder, by Professor Masius (Belgium), it appears that M. Masius, in 1867, demonstrated that the spinal cord cut at the level of the intervertebral disk which separates the sixth and seventh lumbar vertebræ, induced in rabbits a relaxation of the sphincter and a loss of its reflex contractility; and, likewise, that by destroying the spinal cord immediately anterior to this point, and at different distances from it, the sphincter ani, far from relaxing, remains contracted, and even more strongly, and also manifests more vigorous reflex contractility; he has, likewise, determined that a section made below the disk aforesaid induces inverse effects. He has hence concluded the existence in rabbits of a centre (ano-spinal centre) placed in the cord at a point corresponding to union of the sixth and seventh lumbar vertebræ. He has assigned to it the function of controlling the tonic as well as the reflex contraction of the sphincter muscle of the anus. We admit that inhibitory (empêchantes) fibres reach this centre, since the section of those fibres which unite it with the cerebral nervous system increases the tonic and reflex contractions of the sphincter.

Since the publication of this memoir he has extended his experiments to dogs.

It results from these new experiments:

1st. That there exists, in the spinal cord of dogs a centre (ano-spinal centre) situated about the level of the middle and lower third of the fifth lumbar vertebra.

2d. That this centre controls, as in rabbits, the tonic and reflex contractions of the sphincter ani.

3d. That inhibitory or intercepting fibres reach this ano-spinal centre, since every section made in advance of this exaggerates the tonic and reflex contractions.

These experiments prove, likewise, that the ano-spinal centre of dogs is placed higher, that is to say, more in advance than the same centre in rabbits, in which it is located at the level of the intervertebral disk of the sixth and seventh lumbar vertebræ.

It should be remarked that the spinal cord is relatively shorter in dogs than in rabbits; in the former it ceases at the union of the seventh lumbar and the first sacral vertebra. It might then be supposed that in man, in whom the spinal cord is prolonged only to the end of the first lumbar vertebra, if there be an ano-spinal centre it must be situated in the dorsal region. Moreover, this seems demonstrated by an observation reported by M. Gluge. He detected, as a consequence of a fracture of the vertebral column in the region of the sixth dorsal vertebra, relaxation, and loss of tonicity of the sphincter ani, but, likewise, the preservation during a month, of the reflex mobility of this muscle.

These observations warrant the inference, as M. Schwann, our illustrious master, asserts, that there exist, in man, two separate centers: one controlling the tonic, the other the reflex movement of the sphincter ani. But, as may have been perceived, from the investigations referred to above, it has always been observed, as well in rabbits as in dogs, that the same restricted portion of the cord holds under its control both the reflex and tonic mobility of the orbicular muscle of the anus. Hence the observations of M. Gluge appear insufficient to prove the existence, in man, of two separate centers.

Important investigations have been made, recently, to determine the influence of the nervous system upon the bladder. *Budge attempted to demonstrate that the cerebral peduncles furnished motor fibres to the bladder, fibres which traverse the anterior columns of the cord, and the third and fourth sacral nerves. These fibres transmit voluntary impulses to the bladder. A second source of motor fibres, disconnected with the act of volition, exists in the lumbar portion of the cord. Giannuzzi and Budge determined, by experiments made upon dogs, that two principal points of the spinal cord control the contractions of the bladder; one corresponding to the third and the other to the fifth lumbar vertebra.

The fibres which separate from the spinal cord, at the level of the these lumbar vertebra, pass by the cord, and the mesenteric ganglia of the great sympathetic, to reach the hypogastric plexus.

The fibres which originate at the fifth lumbar vertebra reach the hypogastric plexus directly by the sacral nerves. The sensory nerves of the bladder, which transmit reflex excitation to the spinal cord, are contained in the hypogastric plexus; they reach the spinal cord through the anastomotic branches of this plexus, with the trunk of the lumbar sympathetic, by communicating branches which unite this last with the cord, and, lastly, by the posterior lumbar roots.

This much was known of the action of the nervous system upon the bladder. From the experiments which M. Masias has made upon this subject, he thinks the following conclusions may be drawn:

---

*Constatts Jahresbericht über die Leistungen in den Physiologischen Wissenschaften in Jahre, 1864. S. 237.

1st. Every section of the spinal cord made above the ano-spinal center, induces an exaggeration of the persistent contraction of the sphincter vesicæ.

2d. Every section performed a little lower induces a relaxation of this muscle.

3d. The tonic and the reflex motility of the sphincter are under the control of the same portion of the cord, which is situated, in rabbits, at the union of the middle with the lower third of the seventh lumbar vertebra; and in dogs, on a level with the lower portion of the fifth lumbar vertebra.

4th. Another center (vesico-spinal) must be admitted, below the ano-spinal center, (*) which, although very near the first is perfectly distinct from it.

Before concluding, the author adds, the analogy existing between these two centers ano-spinal and vesico-spinal ought to be indicated: the first controls the tonic and reflex contractility of the anal sphincter; the second regulates the tonic and reflex contractions of the sphincter of the bladder.

We have determined that inhibitory (empêchantes) fibres reach the anal center; and analogous fibres extend to the vesico spinal center, for, when the fibres which maintain the communication of this center with the cerebral system are cut, the tonic and reflex motility of the vesical spincter are augmented.

---

*The center termed here vesico-spinal must not be confounded with genito-spinal, of Budge; this last center regulates the involuntary contractions of the bladder; the first controls the tonic and reflex contractions of the sphincter of the bladder.—*Masius.*

